# Current practice patterns of preoperative bowel preparation in colorectal surgery: a nation-wide survey by the Chinese Society of Colorectal Cancer

**DOI:** 10.1186/s12957-018-1440-4

**Published:** 2018-07-09

**Authors:** Zheng Liu, Ming Yang, Zhi-xun Zhao, Xu Guan, Zheng Jiang, Hai-peng Chen, Song Wang, Ji-chuan Quan, Run-kun Yang, Xi-shan Wang

**Affiliations:** Department of Colorectal Surgery, National Cancer Center/National Clinical Research Center for Cancer/Cancer Hospital, Chinese Academy of Medical Sciences and Peking Union Medical College, Beijing, China

**Keywords:** Bowel preparation, Mechanical bowel preparation, Colorectal surgery, Survey

## Abstract

**Background:**

The optimal preoperative bowel preparation for colorectal surgery remains controversial. However, recent studies have established that bowel preparation varies significantly among countries and even surgeons at the same institution. This survey aimed to obtain information on the current practice patterns of bowel preparation for colorectal surgery in China.

**Methods:**

A paper-based survey was circulated to the members of the Chinese Society of Colorectal Cancer (CSCC). The survey responses were collected and analyzed. Statistical analysis was performed for all the categorical variables according to the responses to individual questions.

**Results:**

Three hundred forty-one members completed the questionnaire. Regarding surgical practice, 203 (59.5%) performed > 50% of the colorectal operations laparoscopically or robotically; the use of mechanical bowel preparation (MBP) alone was significantly higher (63.5 vs 31.9%; *P* < 0.001). The respondents who performed > 200 colonic or rectal resections provided significantly more MBP alone (79.6 vs 39.1%, *P* < 0.001; 76.6 vs 43.2%, *P* < 0.001; respectively). Among hospitals with fewer than 500 beds, 52.4% of the respondents used MBP + oral antibiotics preparation (OAP) + enema, a significantly higher percentage than the respondents of hospitals with more than 500 beds (*P* < 0.001). Nearly 40% of the respondents prescribed OAP in regimens; meanwhile, 74.8% prescribed preoperative intravenous antibiotics.

**Conclusions:**

The study demonstrates considerable variation among members from the CSCC. These findings should be considered when developing multicenter trials and to provide more definitive answers.

**Electronic supplementary material:**

The online version of this article (10.1186/s12957-018-1440-4) contains supplementary material, which is available to authorized users.

## Background

Although preoperative bowel preparation is a standard practice for the most elective colorectal surgical procedures and is routinely used, the method and practice still vary widely [1–3]. In the past few decades, various regimens of mechanical bowel preparation (MBP) and oral antibiotics preparation (OAP) have been widely debated [4–7]. Previous investigators have suggested that MBP or OAP reduces the risk of anastomotic leaks and infectious complications [8, 9]. It is widely accepted that MBP/OAP could help to reduce the stool burden and further reduce the bacterial counts [10, 11].

A recent study has added fuel to this debate. The analysis of the American College of Surgeons National Surgical Quality Improvement Program (ACS-NSQIP) indicated that the combined use of MBP/OAP was associated with significantly lower rates of postoperative complications compared with the use of other bowel preparation strategies [12]. However, a multicenter randomized trial of 1354 patients found that performing colorectal surgery safely without MBP was justified [13]. This is in keeping with common belief that clinical practice is not always evidence-based but is based on tradition and an individual’s opinion and previous experiences [14].

Although optimal bowel preparation remains elusive, understanding these differences in practice can help continually improve the clinical practices and implement multicenter trials. To the best of our knowledge, no such survey of preoperative bowel preparation has been previously undertaken in China. The purpose of this study was to describe the current practice patterns of preoperative bowel preparation in colorectal surgery among members of the Chinese Society of Colorectal Cancer (CSCC).

## Methods

A 19-question paper-based survey was developed (see Additional file 1). The permission to conduct the survey was obtained from the CSCC. The anonymous survey was announced by posters to the active members who attended the Annual Meeting of the CSCC on August 18–20, 2017. The participants could complete the questionnaire immediately before, during, or after the meeting, depending on their individual needs and predilections. Participation was encouraged by the program coordinators but was not mandatory.

Key demographic information was collected, including gender, age, experience time, medical specialty, affiliations, position, and volume. Specific questions were aimed at the methods and practices used for preoperative bowel preparation in colorectal surgery in the respondent’s practice. The survey consisted of questions regarding the use of MBP, OAP, and perioperative intravenous antibiotics for colorectal surgery. We also asked for information on whether the respondents had used bowel preparation in incomplete bowel obstruction.

Based on the responses obtained, the response rates of respondents were calculated; Fisher’s exact test analysis was used to compare groups using SPSS (version 19.0; IBM Corporation, Armonk, NY).

## Results

### Demographics

Overall, 341 members finally completed the questionnaire, representing 31 provincial administrative regions. Table 1 shows the demographic characteristics of the respondents. There were 318 (93.3%) male respondents and 23 (6.7%) female respondents. Most of the respondents had more than 10 years of working experience (71.3%), and working in general hospitals (86.5%), and were under the age of 40 (57.8%). The most common specialty for the respondents was general surgery (49.6%), and 38.4% reported working in hospitals with more than 1500 beds. Regarding the surgical volume, 28.7% performed > 200 colonic resections per year and 56.9% performed < 100 rectal resections per year. Among the respondents, 59.5% performed > 50% of colorectal operations laparoscopically or robotically.

**Table 1:** General characteristics

	Number	Percent
Gender		
Male	318	93.3
Female	23	6.7
Age		
< 40 years	197	57.8
40–50 years	121	35.5
> 50 years	23	6.7
Working experience		
< 10 years	98	28.7
10–20 years	155	45.5
> 20 years	88	25.8
Medical specialty		
General surgery	169	49.6
Gastrointestinal surgery	81	23.8
Colorectal surgery	50	14.7
Other	41	12.0
Hospital setting		
General	295	86.5
Specialized	46	13.5
Hospital volume		
< 500 beds	63	18.5
500–1000 beds	87	25.5
1000–1500 beds	60	17.6
> 1500 beds	131	38.4
Colonic resections per year		
< 100	181	53.1
100–200	62	18.2
> 200	98	28.7
Rectal resections per year		
< 100	194	56.9
100–200	70	20.5
> 200	77	22.6
Resection performed laparoscopically or robotically		
< 30%	76	22.3
30–50%	62	18.2
> 50%	203	59.5

### Bowel preparation strategies

For colorectal surgery, all the respondents routinely used preoperative bowel preparation. Approximately half of the respondents used MBP alone; MBP + OAP was used by 16.1%, and MBP + OAP combined with an enema (MBP + OAP +enema) was used by 23.8% (Table 2). No respondent used OAP alone. Enema alone and other regimens were prescribed preoperatively by 5.9 and 3.5%, respectively. The percentage of the respondents performing preoperative bowel preparation for colonic resection only or rectal resection only was 2.6 and 10.3%, respectively. Moreover, 71.3% of the respondents reported using bowel preparation for intestinal obstruction patients.

**Table 2:** Answers according to bowel preparation

	Number	Percent
Bowel preparation regimens		
MBP alone	173	50.7
MBP + OAP + enema	81	23.8
MBP + OAP	55	16.1
Enema alone	20	5.9
Other	12	3.5
Indication for bowel preparation		
Colonic resection only	9	2.6
Rectal resection only	35	10.3
Colonic resection + rectal resection	297	87.1
Bowel preparation for intestinal obstruction		
Yes	243	71.3
No	98	28.7
Preoperative intravenous antibiotic		
Yes	255	74.8
No	86	25.2
Postoperative intravenous antibiotic		
Yes	307	90.0
No	34	10.0
Length of postoperative intravenous antibiotic usage		
< 1 days	14	4.6
1–3 days	125	40.7
> 3 days	168	54.7

*MBP* mechanical bowel preparation, *OAP* oral antibiotics preparation

**Fig. 1:**
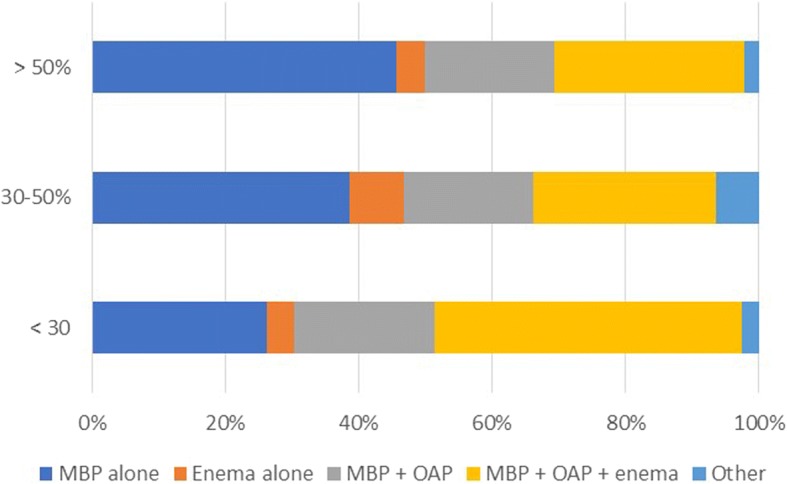
Association between percentages of resections performed laparoscopically or robotically and bowel preparation. MBP mechanical bowel preparation; OAP oral antibiotics preparation

**Fig. 2:**
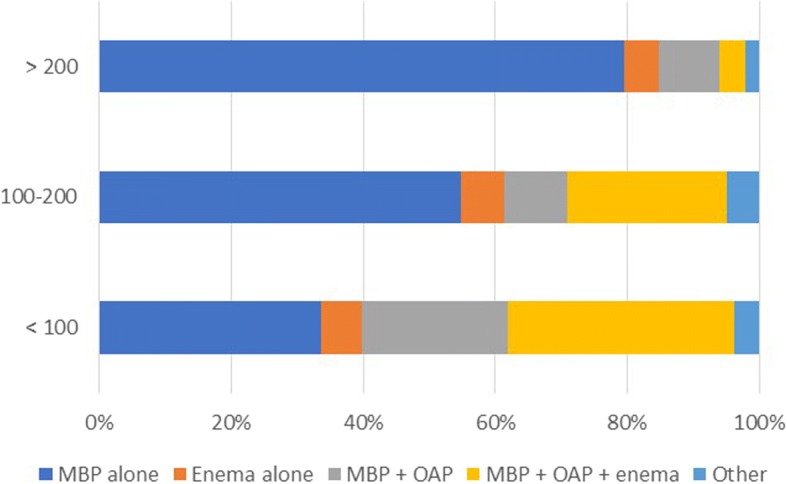
Association between colonic resections per year and bowel preparation. MBP mechanical bowel preparation; OAP oral antibiotics preparation

**Fig. 3:**
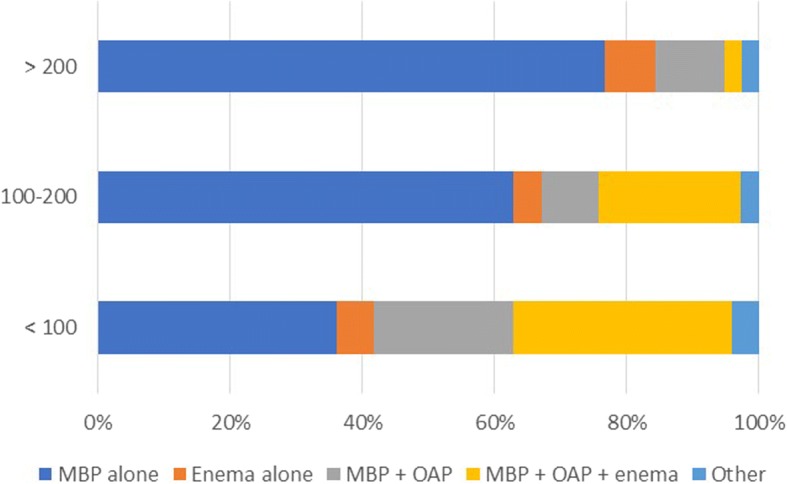
Association between rectal resections per year and bowel preparation. MBP mechanical bowel preparation; OAP oral antibiotics preparation

**Fig. 4:**
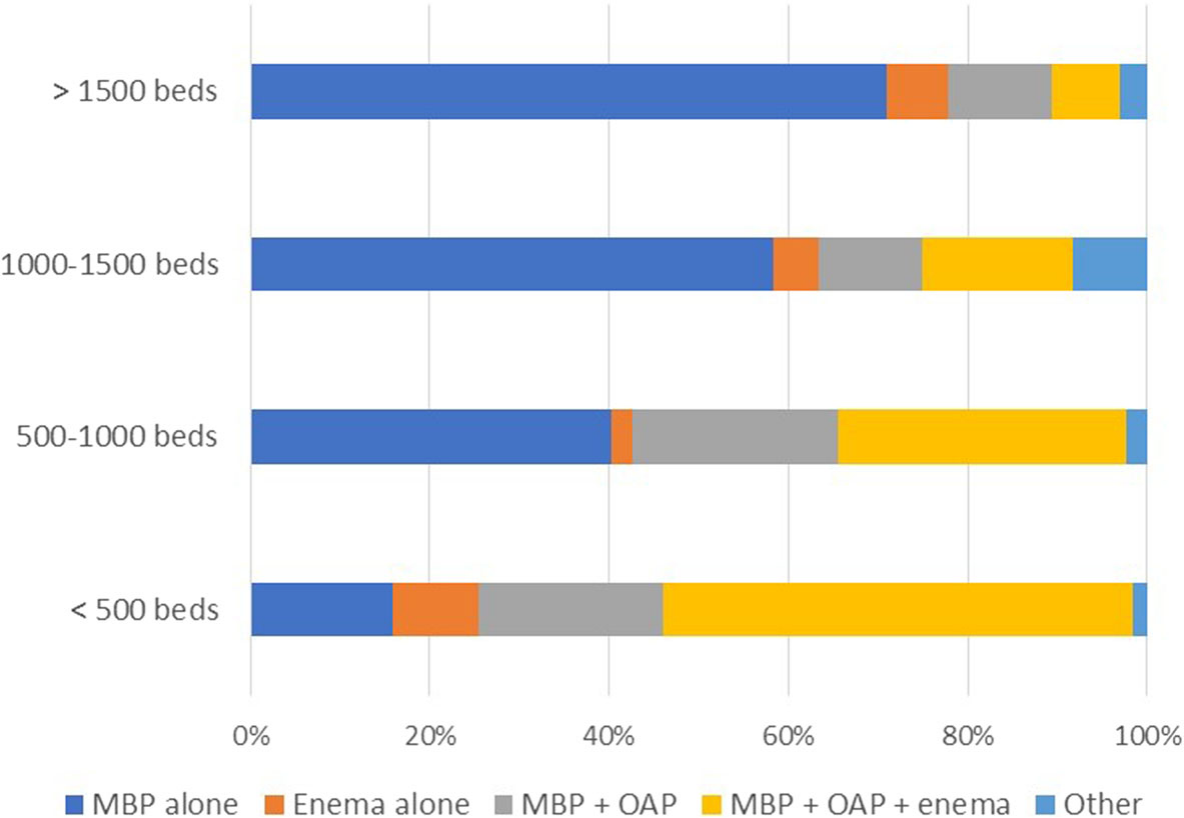
Association between hospital volume and bowel preparation. MBP mechanical bowel preparation; OAP oral antibiotics preparation

The respondent’s age, hospital volume, volume of resections per year, and percentage of resections performed laparoscopically or robotically showed significant differences in the use of preoperative bowel preparation (Table 3). In the cohort performing > 50% of colorectal operations laparoscopically or robotically (*n* = 203), the use of MBP alone was significantly higher (63.5 vs 31.9%; *P* < 0.001) (Fig. 1). The respondents who performed > 200 colonic or rectal resections gave significantly more MBP alone (79.6 vs 39.1%, *P* < 0.001; 76.6 vs 43.2%, *P* < 0.001; respectively) (Figs. 2 and 3). Of hospitals with less than 500 beds, 52.4% of the respondents used MBP + OAP + enema, which is significantly higher than the respondents of hospitals with more than 500 beds (*P* < 0.001) (Fig. 4). The respondent’s working experience and hospital setting did not significantly affect the use of bowel preparation.

**Table 3:** Subgroup analysis of preoperative bowel preparation use

						*P* value
	MBP alone	Enema alone	MBP + OAP	MBP + OAP + enema	Other	
Age						
< 40 years	101	16	22	51	7	0.032
40–50 years	60	2	27	27	5	
> 50 years	12	2	6	3	0	
Working experience						
< 10 years	55	8	8	24	3	0.128
10–20 years	76	9	26	36	8	
> 20 years	42	3	21	21	1	
Hospital setting						
General	148	16	49	72	10	0.738
Specialized	25	4	6	9	2	
Hospital volume						
< 500 beds	10	6	13	33	1	< 0.001
500–1000 beds	35	2	20	28	2	
1000–1500 beds	35	3	7	10	5	
> 1500 beds	93	9	15	10	4	
Colonic resections per year						
< 100	61	11	40	62	7	< 0.001
100–200	34	4	6	15	3	
> 200	78	5	9	4	2	
Rectal resections per year						
< 100	70	11	41	64	8	< 0.001
100–200	44	3	6	15	2	
> 200	59	6	8	2	2	
Resection performed laparoscopically or robotically						
< 30%	20	3	16	35	2	< 0.001
30–50%	24	5	12	17	4	
> 50%	129	12	27	29	6	

*MBP* mechanical bowel preparation, *OAP* oral antibiotics preparation

### OAP and intravenous antibiotics

Preoperative oral antibiotics were administered by 39.9% of the respondents. The most common specified antibiotic drug used was metronidazole (83.9%). Preoperative or postoperative intravenous antibiotics were administered by most respondents (74.8 vs 90.0%, respectively). The length of postoperative usage was < 1 day in 4.6%, 1–3 days in 40.7%, and > 3 days in 54.7% of the respondents.

## Discussion

For several decades, surgeons have utilized bowel preparation to reduce infectious complications, but the value has remained controversial. The current survey is the first nation-wide attempt to document the current trends of preoperative bowel preparation in China. Among the respondents who were older, were working in a large volume hospital, and were performing a higher percentage of minimally invasive surgeries, a significantly higher use of MBP alone was noted. This study observed variations in bowel preparation across respondents from CSCC.

The use of MBP in elective colorectal surgery is supported by emerging evidence, although several published randomized controlled trials have shown that preoperative MBP should be omitted before colon surgery [13, 15, 16]. There is ongoing debate on the role of bowel preparation in colorectal surgery, MBP is still used in routine clinical practice before both colon and rectal surgery in China, with a similar picture in the USA and Japan [17–19]. Unlike European practice, American-enhanced recovery guidelines often include MBP [20]. Why is this discrepancy evident between American and European guidelines? One possible reason may be that the European recommendation is not to be revisited at present [1].

The 2017 clinical practice guidelines from the American Society of Colon and Rectal Surgeons (ASCRS) and Society of American Gastrointestinal and Endoscopic Surgeons (SAGES) recommend MBP + OAP before colorectal surgery as preferred preparation to reduce complication rates [21]. Surveys have shown a change in the use of laparoscopic procedures compared with open procedures depending on the type of preparation used. A survey from the European Society of Coloproctology (ESCP) found that the routine use of MBP prescribed by laparoscopic surgeons was significantly lower (19.7 vs 51.5%, *P* < 0.01) [22]. By contrary, a survey from the UK showed that a higher proportion of laparoscopic right-sided procedures was performed with MBP compared with open procedures (16.8 vs 9.5%; *P* = 0.08); however, the need for MBP for a left-sided procedure remains controversial [23]. Despite the survey limitation of unclear procedure classification in the questionnaire, this study showed a similar picture of the high use of MBP in laparoscopic or robotic surgery. Although previous studies have suggested that MBP did not improve postoperative outcomes in laparoscopic colorectal resections [24], there is an inconsistency between opinion and practice, with individual surgeons often using different regimens for their open and laparoscopic resections [23].

OAP is generally believed to help protect against infectious complication in elective colorectal resections [25]. Currently, it is becoming increasingly clear that MBP + OAP combined with intravenous antibiotics is the most effective method. Previous surveys from the USA, Europe, and Japan have shown a low rate of oral antibiotic usage [17–19, 22]. This obviously contrasts with the patterns of practice in China, because nearly 40% of the respondents prescribed OAP in regimens, meanwhile 49.3% prescribed a longer duration (> 3 days) of postoperative intravenous antibiotics. Our results showed that, despite the clear recommendations from the literature and the guidelines, there remains some concern about the overuse of antibiotics in China.

Moreover, in our subgroup analysis, different bowel preparation strategies are associated with hospital volume. Our results may reflect the surgeon’s bias or limitations inherent in this type of survey. Regarding the lower use of OAP, our results showed that, despite the disparity among hospitals, high-volume hospitals tend to follow guidelines more closely. The other interesting finding in our study is that bowel preparation (enema) for intestinal obstruction is common (71.3%). Although enema could stimulate the colon to contract and eliminate stool, it may cause serious adverse events, such as perforation or metabolic derangement [26]. Our findings should lead to a careful consideration of appropriate bowel preparation to intestinal obstruction.

## Conclusions

In conclusion, this survey provides an adequate response from the CSCC members, describing the preoperative bowel preparation in current practices. Regarding the respondent’s age, hospital, and resection volume, as well as the percentage of minimally invasive resections, the study shows that there is no current standardization of preoperative bowel preparation among colorectal surgeons in China, especially concerning the use of oral or intravenous antibiotic prophylaxis. Therefore, we recommend the CSCC should use these results to develop new protocols for multicenter trials and provide more definitive answers.

## Additional file

Additional file 1: A 19-question paper-based survey. (DOCX 18 kb)

## Abbreviations

ACS-NSQIP: American College of Surgeons National Surgical Quality Improvement Program
ASCRS: American Society of Colon and Rectal Surgeons
CSCC: Chinese Society of Colorectal Cancer
ESCP: European Society of Coloproctology (ESCP)
MBP: Mechanical bowel preparation
OAP: Oral antibiotics preparation
SAGES: Society of American Gastrointestinal and Endoscopic Surgeons
