# Supplementary material for: Current practice patterns of preoperative bowel preparation in colorectal surgery: a nation-wide survey by the Chinese Society of Colorectal Cancer
Source: World J Surg Oncol. 2018 Jul 9;16:134. doi: 10.1186/s12957-018-1440-4 (PMC6038260; doi:10.1186/s12957-018-1440-4)
Supplement: Supplementary file 1 — A 19-question paper-based survey. (DOCX 18 kb) [file 12957_2018_1440_MOESM1_ESM.docx]

1. What is your gender?

○ Male

○ Female

2. What is your age?

○ <40 years

○ 40-50 years

○ >50 years

3. How many years of your work experience?

○ <10 years

○ 10-20 years

○ >20 years

4. In which of the following hospital do you practice?

○ General

○ Specified

5. How many beds of your hospital?

○ <500

○ 500-1000

○ 1001-1500

○ >1500

6. Your medical specialty?

○ General surgery

○ Gastrointestinal surgery

○ Colorectal surgery

○ Other

7. Number of colonic resections you perform per year?

○ <100

○ 100-200

○ >200

8. Number of rectal resections you perform per year?

○ <100

○ 100-200

○ >200

9. Percentage of colorectal operations performed laparoscopically or robotically?

○ <30%

○ 30-50%

○ >50%

10. Which of the following do you prescribe preoperatively?

○ None

○ MBP only

○ Enema only

○ OAP + MBP

○ OAP + MBP + enema

○ Other

11. If you use oral antibiotic, which antibiotic do you prescribe?

○ Metronidazole

○ Gentamicin

○ Streptomycin

○ Norfloxacin

○ Other

12. If you use oral antibiotic, for how many days pre-operatively?

○ 1 day

○ 2 days

○ 3 days

13. For which of the following operations do you prescribe bowel preparation preoperatively?

○ Colonic or rectal resection

○ Colonic only

○ Rectal only

14. Do you consider bowel preparation preoperatively for laparoscopic surgery?

○ Yes

○ No

15. Do you consider bowel preparation preoperatively for incomplete intestinal obstruction?

○ Yes

○ No

16. Which of the following do you prescribe?

○ MBP only

○ Enema only

○ OAP + MBP

○ OAP + MBP + enema

○ other

17. Do you prescribe intravenous preoperative antibiotics?

○ Yes

○ No

18. Do you prescribe intravenous postoperative antibiotics?

○ Yes

○ No

19. For how many days postoperatively intravenous antibiotics?

○ >3 days

○ 1-3 days

○ <1 day
